# Trypanosomiasis-Induced B Cell Apoptosis Results in Loss of Protective Anti-Parasite Antibody Responses and Abolishment of Vaccine-Induced Memory Responses

**DOI:** 10.1371/journal.ppat.1000078

**Published:** 2008-05-30

**Authors:** Magdalena Radwanska, Patrick Guirnalda, Carl De Trez, Bernard Ryffel, Samuel Black, Stefan Magez

**Affiliations:** 1 Laboratoire de Parasitologie, Université Libre de Bruxelles, ULB, Brussels, Belgium; 2 Department of Veterinary and Animal Sciences, University of Massachusetts, Amherst, Massachusetts, United States of America; 3 Molecular Immunology and Embriology IEM2815, Centre National de la Recherche Scientifique, Orléans, France; 4 Laboratory for Cellular and Molecular Immunology, Vrije Universiteit Brussel, Brussels, Belgium; 5 Department of Molecular and Cellular Interactions, VIB, Brussels, Belgium; London School of Hygiene and Tropical Medicine, United Kingdom

## Abstract

African trypanosomes of the *Trypanosoma brucei* species are extra-cellular parasites that cause human African trypanosomiasis (HAT) as well as infections in game animals and livestock. Trypanosomes are known to evade the immune response of their mammalian host by continuous antigenic variation of their surface coat. Here, we aim to demonstrate that in addition, trypanosomes (i) cause the loss of various B cell populations, (ii) disable the hosts' capacity to raise a long-lasting specific protective anti-parasite antibody response, and (iii) abrogate vaccine-induced protective response to a non-related human pathogen such as *Bordetella pertussis*. Using a mouse model for *T. brucei*, various B cell populations were analyzed by FACS at different time points of infection. The results show that during early onset of a *T. brucei* infection, spleen remodeling results in the rapid loss of the IgM^+^ marginal zone (IgM^+^MZ) B cell population characterized as B220^+^IgM^High^IgD^Int^ CD21^High^CD23^Low^CD1d^+^CD138^−^. These cells, when isolated during the first peak of infection, stained positive for Annexin V and had increased caspase-3 enzyme activity. Elevated caspase-3 mRNA levels coincided with decreased mRNA levels of the anti-apoptotic Bcl-2 protein and BAFF receptor (BAFF-R), indicating the onset of apoptosis. Moreover, affected B cells became unresponsive to stimulation by BCR cross-linking with anti-IgM Fab fragments. *In vivo*, infection-induced loss of IgM^+^ B cells coincided with the disappearance of protective variant-specific T-independent IgM responses, rendering the host rapidly susceptible to re-challenge with previously encountered parasites. Finally, using the well-established human diphtheria, tetanus, and *B. pertussis* (DTPa) vaccination model in mice, we show that *T*. *brucei* infections abrogate vaccine-induced protective responses to a non-related pathogen such as *B. pertussis*. Infections with *T. brucei* parasites result in the rapid loss of T–cell independent IgM^+^MZ B cells that are normally functioning as the primary immune barrier against blood-borne pathogens. In addition, ongoing trypanosome infections results in the rapid loss of B cell responsiveness and prevent the induction of protective memory responses. Finally, trypanosome infections disable the host's capacity to recall vaccine-induced memory responses against non-related pathogens. In particular, these last results call for detailed studies of the effect of HAT on memory recall responses in humans, prior to the planning of any mass vaccination campaign in HAT endemic areas.

## Author Summary

African trypanosomes are extracellular parasites that cause the deadly disease sleeping sickness in humans, and nagana in cattle. The control of infection is believed to be largely dependent on the host antibody response. We postulate here that protective anti-trypanosome responses mainly involve splenic marginal zone B cells, as they are implicated in the production of antibodies against blood-borne pathogens. In this work, we show that trypanosome infections induce the rapid loss of these marginal zone B cells, coinciding with the loss of the splenic marginal zone itself. While the infection does result in the induction of plasma cell differentiation and antibody secretion, the loss of the marginal zone B cell population results in the loss of specific protective responses. In addition, we also show that host memory responses are destroyed during infection, even affecting unrelated vaccine-induced memory responses such as those induced by the commercially available DTPa vaccine. The latter finding is crucial for the evaluation of mass vaccination approaches in African regions where trypanosome infections are prevalent.

## Introduction

African trypanosomes that belong to the *T. brucei* species are extracellular parasites that cause Human Afican Trypanosomiasis (HAT) and Nagana, a wasting disease of cattle. As a defense barrier against the host immune response, the entire surface of the *T. brucei* parasite is covered with 10^7^ densely packed molecules of a variant surface glycoprotein (VSG) that determines the antigenic phenotype of the parasite [1],[2],[3]. At any given time, a single VSG gene encodes for all the VSG molecules present on the trypanosome surface, creating a homogenous antigenic coat. There are at least 1000 different VSG genes present within the *T. brucei* genome [3],[4],[5]. In addition, these VSG genes undergo extensive recombination generating an extremely large and plastic antigenic repertoire. The ability to switch expression from one VSG to another is considered to be the major mechanism allowing the parasite to evade an efficient host antibody response, hence preventing parasite elimination and permitting the establishment of a chronic infection [3],[6]. In addition, the extreme degree of antigenic variation exhibited by African trypanosomes is considered to be the main reason for the failure of anti-trypanosome vaccination strategies to date [7].

Experimental *T. brucei* infections in mice are widely used to study host pathogen interactions [8],[9], and serve as models for anti-trypanosome vaccine development [10]. These infections are characterized by the recurring appearance of peaks of parasitemia corresponding to the newly emerging variant antigenic types (VATs) of the parasite. Parasite elimination from the blood, lymph and various host tissues can result from combined antibody-mediated killing, nitric oxide and cytokine toxicity, and parasite growth arrest in response to host and parasite-derived quorum-sensing factors [9],[11],[12],[13],[14],[15]. During the first days of infection, mice generate a rapid IgM response, followed by an immunoglobulin isotype switch and secretion of high levels of IgG2a, IgG2b and IgG3 antibodies [16]. Although infections trigger both a T-dependent and a T-independent antibody response, host resistance was shown to be mainly dependent on the latter and functions through a complement independent mechanism. Indeed, trypanosome infected athymic mice as well as complement-deficient mice mount an effective antibody response and control parasite growth with similar kinetics as observed in wild-type mice [16],[17],[18]. Interesting to note is that while early induction of anti-trypanosome antibody responses aids in the effective VAT-specific clearance of the first peak of parasitemia, mice lose the capacity to control *T. brucei* growth later during infection, and usually die due to multi-organ failure with high circulating parasite loads in the presence of high levels of anti-VSG antibodies [16],[18]. Interestingly, while polyclonal B cell activation was found to be a hallmark of the infection with human infective parasites [19],[20] and in trypanosome infected domestic animals [21], the mechanisms underlying this process are still not fully understood. It has been proposed that early on during infection, the densely packed VSG coat can be recognized as a highly repetitive and structured single-epitope array implicated in abnormal B cell activation as well as exhaustion [22].

To date, little is known about the fate of specific B cell populations and the tissue re-modeling during trypanosome infections, although early studies have reported changes in splenic B cell content indicating high cellular infiltration into the spleen. In general, the earliest B cells that migrate from bone marrow (BM) to the spleen are transitional type 1 (T1) B cells. These cells develop locally into transitional type 2 (T2), and next into marginal zone (MZ) B cells or mature follicular (Fo) B cells. MZ B cells play an important role in the early phases of antibody responses against mainly T-independent antigens [23],[24],[25]. As T-independent B cell responses are crucial for early control of *T. brucei*, the role and fate of MZ B cells was further analyzed here. Splenic MZ B cells can be characterized as B220^+^IgM^High^IgD^Int^CD21^High^CD23^Low^CD1d^+^CD138^−^ [26]. Overall, B cell differentiation and survival is regulated by the B cell-activating factor (BAFF), a member of the TNF family. BAFF-R, one of the three BAFF receptors, is expressed on B cells and controls overall B cell homeostasis [27]. The binding of the BAFF to BAFF-R promotes ΝF-κB activation and increases mRNA levels of the anti-apoptotic factor Bcl-2 [28]. Despite these data, and despite the fact that *T. brucei* infections were reported to induce B cell unresponsiveness to mitogenic stimuli [29],[30],[31], trypanosome-induced B cell apoptosis has not received major attention so far. In general, apoptosis occurs in several pathological and non-pathological conditions and constitutes a part of a mechanism of cell replacement and tissue re-modeling, leading to maintenance of cellular homeostasis [32],[33]. The apoptotic process is characterized by the series of morphological changes such as cell shrinkage, chromatin condensation, and DNA fragmentation. Here, the family of caspases plays a central role, with the activation of caspase-3 by the release of cytochrome C regarded as a primary mechanism involved in apoptosis and the degradation of chromosomal DNA [34]. The anti-apoptotic Bcl-2 protein counteracts this process by inhibiting the release of cytochrome C, which in turn blocks activation of caspase-3 [35].

Given the crucial role of T-independent IgM responses in trypanosomiasis control, and the lack of data on the fate of B cell populations and in particular IgM^+^MZ B-cells during infection, this paper focuses on this rather neglected part of the host immune response.

Here we demonstrated that extensive remodeling of spleen micro-architecture takes place early after *T. brucei* infection. This event coincides with the drastic reduction in IgM^+^MZ B cells. Moreover, the loss of IgM^+^ B cells after *in vivo* infection rendered mice susceptible to a challenge with a previously encountered *T. brucei* variant antigenic type. Together, our data indicates that while antigenic variation might aid in perpetuating a *T. brucei* infection within a given host, active infection-driven elimination of IgM^+^ B cells renders a host susceptible to repetitive infections by the same antigenic type trypanosome. Moreover, in this paper we demonstrated that *T. brucei* infection can also abrogate vaccine induced protective responses that were generated against non-related pathogens such as *Bordettela pertussis* (*B. pertussis*), using a human vaccine against diphtheria, tetanus and *B. pertussis* (DTPa) in a mouse vaccination model.

## Materials and Methods

### Parasites and infection in mice

The pleomorphic AnTat 1.1E (EATRO 1125 stock) *T. brucei brucei* was used in this study as previously described [12]. This infection is characterized by a multi-wave parasitemia development, in which every wave represents a parasite population of different antigenic type. Mice were infected by i.p. injection of 5000 parasites/mouse. Every 2 to 3 days, the number of parasites present in the blood was estimated using a counting chamber and a light microscope. For re-challenge experiments, cloned monomorphic *T. b. brucei* AnTat 1.1E or control monomorphic *T. b. brucei* MITat 1.4 parasites were used. These clones are characterized by the rapid killing of their host, expressing one single VAT and their lack of antigentic variation during the 4 day infection. The homogenic expression of AnTat 1.1 VSG and MITat 1.4 VSG was verified here by RT-PCR followed by *VSG* sequence analysis. Re-challenge experiment of AnTat 1.1E infected mice was performed on day 10 and 17 by administering 5000 cloned monomorphic parasites/mouse. *T. brucei* infections and re-challenge experiments were performed using the following female mice: Balb/c and C57BL/6 (Harlan), and µMT B cell deficient mice as well as nu/nu mice on the C57BL/6 background (both a kind gift of Prof. B. Ruffel, CNRS). All mice were housed in the Animal Facility under barrier conditions. The appropriate university's ethics committees approved all experimental animal procedures.

### B cell proliferation

B cell proliferation assays were performed using splenic B cells from infected or non-infected mice. Cells from day 10 post infection were purified on CD19 MACS separation columns (Miltenyi Biotec). Aliquots of eluted B cells (5×10^4^/ml) were stimulated with different concentration of LPS or anti-IgM Fab fragments. After 24 hours, cells were pulsed with 1 µCi (^3^H) thymidine (AEC Amersham Uppsala, Sweden) and incubated for a further 18 hours.

### Flow cytometry

B cells were analyzed by flow cytomerty. Spleens were harvested from infected and non-infected mice at different time points of infection. Cell suspensions were prepared in complete RPMI 1640. Non-specific binding sites were blocked for 30 min at 4°C, using ice-cold PBS supplemented with 1% normal rat serum and 2.4G2 the anti-FcγR antibody. After washing twice, cells were stained with the following anti-mouse antibodies: anti-IgM, anti-IgD, anti-CD21, anti-CD23, anti-B220, anti-CD1d, anti-CD138, anti-GL7, anti-PNA, coupled to either FITC, or PE. All antibodies were purchased from BD Biosciences (San Jose, CA). Incubations were conducted for 30 min at 4°C. For analysis of apoptosis, cells were simultaneously stained with Annexin V (BD Bioscience) and 7AAD (BD, Biosciences) according to protocols provided by the manufacturers, in combination with the B-cell markers listed above. Stained cells were analyzed on a FACS-Calibur SE flow cytometer (BD Biosciences). Prior to analysis, PI (propidiun iodide) was added to all cell suspension in order to exclude dead cells from the data acquisition. All data was analyzed using the FlowJo (Tri Star) software package.

### Quantitative Real-Time PCR

Gene expression levels were measured by Quantitative Real Time PCR, using the Roche/SYBR green system. B cells from infected and non-infected mice were purified using CD19 magnetic beads. Total mRNA was extracted using Trizol reagent according to the instructions supplied by the manufacturer (Invitrogen). Residual DNA was digested using Turbo DNase kit from Ambion. Reverse transcription was performed using protocol supplied by Invitrogen. Real-time PCR reactions were performed according to the Roche protocol, which included data analysis by ‘Fit Points’, normalization against 18S expression, and ‘Standard Curve Analysis’.

The following primer pairs were used for PCR amplifications:

Bcl2 Forward: 5′- TGGCGCAAGCCGGGAGAACA -3′
Bcl2 Reverse: 5′- CCCGGTGCACAGCGGGCATT -3′
Caspase-3 Forward: 5′- CGGATGTGGACGCAGCCAACC
Caspase-3 Reverse: 5′- CCCCGGCAGGCCTGAATGATGA
BAFF-R Forward: 5′- CTCGTCGGTGCCCCCGCACT
BAFF-R Reverse: 5′- GCGTGGCAGGGCGCTGTCTG
18S Forward: 5′- GGGCGTGGGGCGGAGATATGC
18S Reverse: 5′- CGCGGACACGAAGGCCCCAAA

### Western Blot

Spleens were removed from infected (day 7 post-infection) and non-infected mice. CD21^High^CD23^Low^ MZ B cells were sorted using a FACSVantage (BD bioscience) and used for lysate preparation. Cells were re-suspended in ice-cold PBS supplemented with complete protease inhibitors (Roche). Cell suspensions were sonicated 3 times on ice by delivering the impulse for 30 seconds. Cellular lysate was spun down at 100.000×g for 30 min at 4°C and a soluble fraction was collected. Protein concentration was determined by BCA colorimetric assay (Pierce). 5 µg of soluble proteins were submitted to reducing conditions and separated on SDS PAGE followed by a transfer onto a nitrocellulose. Nitrocellulose membranes were incubated in 1%BSA/Tris pH 8.0 for 1 hour at room temperature in order to block non-specific binding sites. Membranes were washed 5 times with Tris pH 8.0/0.05% Tween 20 solution and incubated with anti-caspase-3 (BD/Pharmingen, clone C92-605, rabbit IgG) antibody diluted 1000 times in 1%BSA/Tris buffer pH 8.0, recognizing mouse pro-caspase 3 as well as the activation cleaved 12 KD and 17 kD caspase 3 products. Next, washed membranes were incubated with a secondary goat anti-rabbit IgG biotinylated antibody (30,000 diluted in Tris buffer) for 1 hour at room temperature. After a washing step, streptavidine/alkaline phosphatase (Sigma) was added to the membranes and incubated for one hour at room temperature. Proteins were visualized by using BCIP/NBT substrate solution from Sigma according to the manufacturer instructions.

### Immunohistochemistry

Spleens were removed from control and infected mice at day 10 post infection. They were embedded in Tissue-Tek (Sakura Finetek USA, Torrance, California, United States), frozen on dry ice and stored at −80°C. Cryostat sections (6–8-µm thick) were fixed in ice-cold acetone for 10 min, rehydrated in PBS, and treated for 30 min with PBS 1% blocking reagent (PBS-BR) (Boehringer Mannheim, Mannheim, Germany). Obtained cryosections were washed in PBS and stained for 60 min with the following biotinylated-labeled antibodies: MOMA-1 (anti-metallophillic marginal macrophages (MMM)) and ERTR-9 (anti-marginal zone macrophages (MZM) (BMA Biomedicals, Augst, Switzerland) resuspended in PBS-BR buffer. Next, the sections were washed in PBS and incubated for 30 min with the secondary detection reagents streptavidin-FITC and streptavidin-Cy3 (Zymed Laboratories, Invitrogen, Carlsbad, California, United States) in PBS-BR for MZM and MMM visualization, respectively. The slides were washed in PBS and mounted in anti-fading GEL/MOUNT (Biomeda, Foster City, California, United States).

Digitized images were captured using a Zeiss AxioCam color camera and analyzed using the Photoshop software (Adobe Systems, San Jose, California, United States).

### Diphtheria, tetanus and *Bordetella pertussis* (DTPa) vaccination scheme and trypanosome challenge

BALB/c neonatal mice were vaccinated according to the previously published protocol [36],[37]. In short, neonatal mice were vaccinated with one quarter of a human dose of the commercially available DTPa vaccine (Boostrix®) administered sub-cutaneous (s.c.). After 21 days, mice received a s.c. booster injection with the same amount of vaccine. After a further 14 days mice were infected i.p with 5000 *T. brucei* parasites/mouse. Ten days post-infection, mice received an intranasal dose of 5×10^6^ *B. pertussis bacteria*/mouse (ATCC9797) in 10 µl PBS. Control mice received the intranasal *B. pertussis* challenge in the absence of a trypanosome challenge, or in the absence of vaccination and parasite infection. Lung bacterial load clearance was monitored after 3 hours, and 3, 5, 8 days post-challenge. Mice were sacrificed and whole lungs were isolated and homogenized in 5 ml PBS. Serial 10 fold dilutions were prepared and aliquots of 200 µl were plated onto the Bordet-Gongaou agar plates. The number of colony forming units (CFU's) was counted after 72 hours of incubation at 36°C.

## Results

### *T. brucei* infections induce both an early loss of Marginal Zone (IgM^+^MZ) B cells and an increase in splenic plasma cells

**Figure 1:**
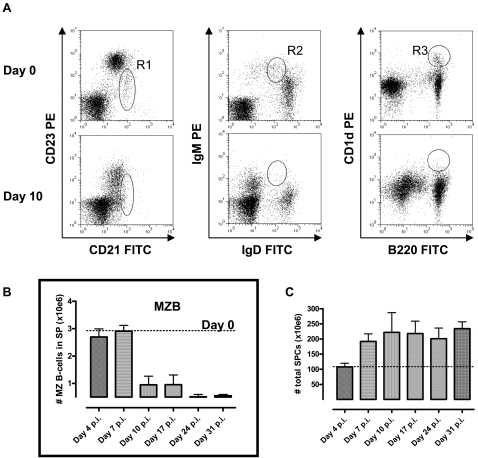
Alterations of Marginal Zone (MZ) B cells during *T. brucei* infections. MZ B cells were detected using FACS as CD21^High^CD23^Low^ (R1), IgD^Int^IgM^High^ (R2) or B220^+^CD1d^+^ (R3) on spleen cells derived from non- infected mice (A, upper FACS panel ) or day 10 *T. brucei* AnTat 1.1E-infected mice (A, lower FACS panel). The decrease in total number of MZ B-cells per spleen was calculated for different time points during infection (B), based on the total amount of cells harvested per spleen at each time point (C). Calculations were performed on cells harvested from 3 individual spleens per time point. Values represent the mean±SD. One of four representative experiments is shown.

**Figure 2:**
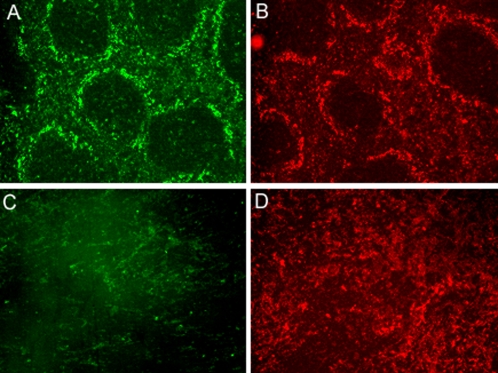
Destruction of spleen marginal zones. The MZ macrophage populations of non-infected (upper panel) and day 10 *T. brucei* AnTat 1.1 infected (lower panel) C57Bl/6 mice are visualized by section staining with ER-TR9 (anti-MZM) (A,C) and MOMA-1 (anti-MMM) antibodies (B,D) (400x magnification).

Induction of a T-independent anti-trypanosome IgM response has been shown to be a crucial factor in *T. brucei* parasite elimination. As IgM^+^MZ B cells are the main mediators of T-independent antigen responses, the fate of this population was addressed in an experimental C57Bl/6 mouse *T. brucei* AnTat 1.1 infection model. Here, the trypanosome infection is characterized by multiple waves of parasitemia and a survival of about 35-40 days [16]. Hence, spleens were isolated at different time points during infection and prepared for cellular characterization by FACS. Multiple surface staining combinations can be used to characterize MZ B cells, with CD21/CD23, IgM/IgD and B220/CD1d as the most common combinations [26]. Figure 1A (upper panel) shows that all three combinations give similar MZ B cell counts when spleen populations of naïve non-infected mice were analyzed, with CD21^High^CD23^Low^ (R1) = 2.25%, IgM^High^IgD^Int^ (R2) = 2.39% and B220^+^CD1d^+^ (R3) = 2.37%. In contrast to naïve spleen populations, MZ B cells were virtually absent from spleen cell populations derived on day 10 of an AnTat 1.1 *T. brucei* infection. Indeed, Fig. 1A (lower panel) shows that all three surface staining combinations indicate a drastic reduction in the % of MZ B cells. In order to calculate the reduction IgM^+^MZ B cell numbers, the R2 gate was used (Fig. 1B). It is important to stress here that during experimental trypanosome infections, a marked splenomegaly takes place [38], resulting in a significant increase by day 10 post-infection of the total amount of cells present within the spleen (Fig. 1C). Hence, in order to incorporate the increase in cell number due to splenomegaly, the % data obtained by FACS was used to obtain the total IgM^+^MZ B cell count *per* spleen. Combined, these data clearly show that even when the increased splenic cellularity of infected mice is taken into account, a significant reduction of splenic IgM^+^MZ B cell numbers takes place right after the first week of infection. In order to visualize the trypanosomiasis-induced loss of the marginal zone, an additional independent strategy was applied. The MZ, delineated by the marginal sinus in the spleen, contained two distinct macrophage populations, marginal metallophilic macrophages (MMM) and marginal zone macrophages (MZM) which localize to the inner and outer rim of the MZ, respectively [39]. By day 10 post-infection, *T. brucei* caused a nearly complete loss of MZM and MMM, identified by the ER-TR9 and MOMA-1 markers, respectively (Fig. 2). Further analysis of the spleen remodeling, revealed that beside the loss of MZ, the entire spleen structure encompassing white and red pulp is lost permanently, confirming previous data [40].

**Figure 3:**
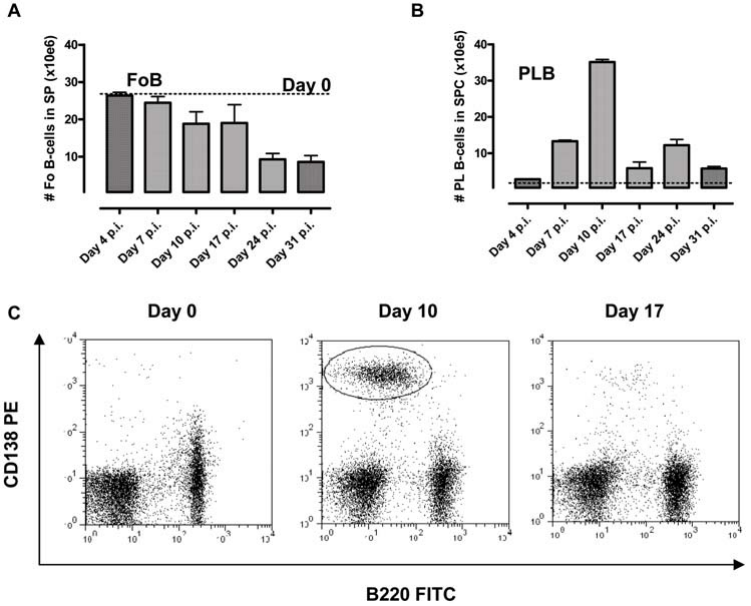
Alteration of Follicular and Plasma B cell numbers during *T. brucei* infections. The number of follicular B cells (A) as well as plasma B cells (B) per spleen was calculated on different days after *T. brucei* AnTat 1.1E infection. Calculations were performed on cells harvested from 3 individual spleens per time point. Values represent the mean±SD. One of four representative experiments is shown. Plasma spleen B cells were stained with a B220/CD138 combination (C).

Besides IgM^+^MZ B cells, splenic B cell populations comprise mainly Follicular (Fo) B cells and plasma (Pl) or germinal center B cells, depending upon the immune status of the host. Given the destruction of the spleen micro-architecture and the absence of induction of germinal center formation during infection, both Fo B and Pl B cell populations were further analyzed in detail by FACS. In naïve mice Fo B cells appear as distinct splenic population expressing CD21^Int^CD23^High^, IgM^Int^IgD ^High^ that distinguishes them from CD21^High^CD23^Low^, IgM^High^IgD^Int^ expressing MZ B cells. They represent up to 25% of the total cell count in a naïve spleen (see Fig 1A). Plotting the alteration in Fo B cell numbers during a *T. brucei* AnTat 1.1 infection shows that also these cell counts decrease towards the end of infection, albeit not with the same magnitude as the MZ B-cells (Fig. 3A). In contrast, when plasma B cells are considered a clear and very significant increase of cell numbers during the first 10 days of infection is observed, that coincided with the disappearance of the MZ B-cells (Fig. 3B). However, these cells rapidly decrease in number again over time, although they always remain significantly increased as compared to naïve plasma spleen B cell counts. Here plasma B cells were characterized by surface expression of B220^+^CD138^+^markers. Figure 3C shows the clear increased percentage of double positive spleen cells on day 10 post-infection, and the relative reduction observed by day 17. When these cells were stained for IgM and CD138, about 50% stained double positive throughout infection, while the others stained IgM^−^CD138^+^ (data not shown).

### *T. brucei* infection induced B cell apoptosis

**Figure 4:**
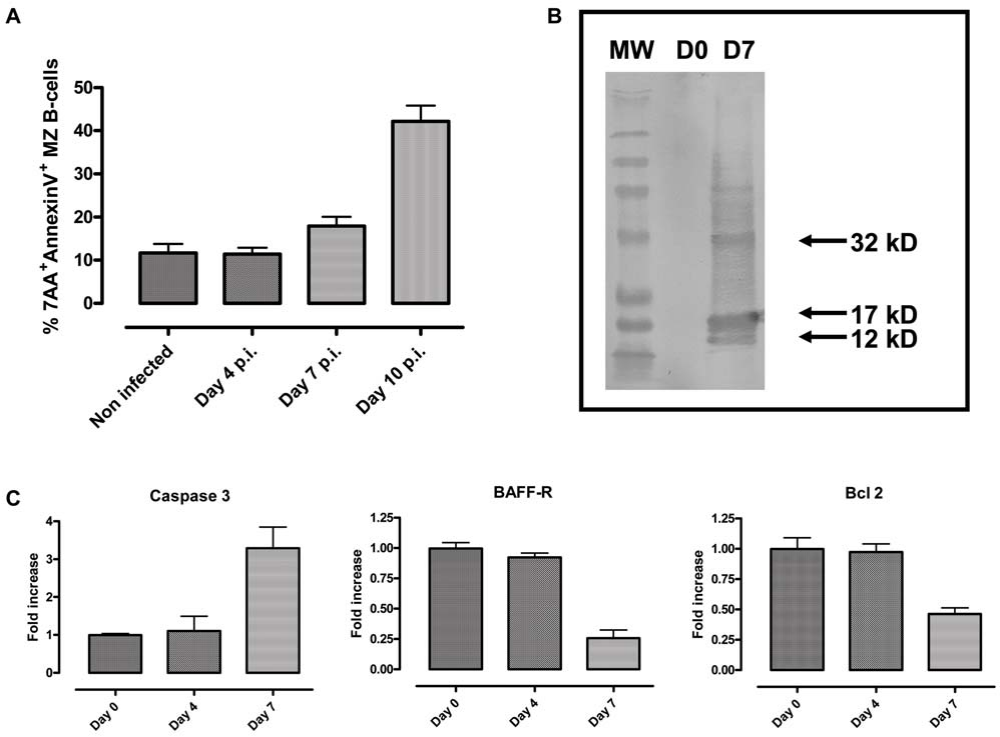
Infection-induced apoptosis of MZB cells. CD21^High^CD23^Low^ cells were gated and analyzed for 7AAD and Annexin V staining. The percentage of positive cells was calculated in MZ B cells from naïve mice, as well as from *T. brucei* AnTat 1.1E infected mice on days 4, 7 and 10 (A). CD21^High^CD23^Low^ cells were FACS sorted on day 7, lyzed and analyzed in Western Blot with an anti-caspase 3 antibody, showing bands at 32 kD (pro-caspase 3), and cleaved forms 17 kD and 12 kD (B). RT-PCR for Casp3, BAFF-R, and Bcl-2 was performed on CD19^+^ spleen cells isolated from naïve mice as well as from *T. brucei-*infected mice on day 4 and day 7 (C). Results present the means of 3 mice per time point ±SD. One of three representative experiments is shown.

The FACS data presented above indicate that MZ B cells are rapidly lost during the first 10 days of a *T. brucei* infection. Besides the possible differentiation of these cells into IgM^+^ plasma cells, we addressed whether apoptosis also contributes to the permanent loss of MZ B-cells. To do so, CD21^High^CD23^Low^ MZ B cells were stained for Annexin V and 7AAD at several time points of the early infection. Figure 4A indicates the induction of apoptosis in this cells population, following the first parasitemia wave and coinciding with the rapid disappearance of the population. In order to independently confirm the induction of infection-associated apoptosis in MZ B cells, CD21^High^CD23^Low^ cells were FACS-sorted and lysed for Western Blot analysis of caspase-3 activation, using a specific antibody recognizing both pro-caspase 3 (32 kDa) and the cleaved caspase 3 bands of 12 and 17 kDa that occur when the apoptosis is taking place. As shown in Fig. 4B, lysate from infection-derived MZ B-cells showed the induced presence of all three caspase 3 forms, while lysates from naïve MZ B cells, loaded on the SDS-PAGE at the same protein concentration, did not score positive for caspase-3 activation.

Additionally, we performed a number of quantitative Real-Time PCR, measuring mRNA levels for caspase-3 as well as mRNA levels coding for the anti-apoptotic protein Bcl-2 and the BAFF receptor (BAFF-R), mainly expressed on B cells and critically involved in B cell homeostasis [28]. Due to the fact that the time consuming FACS sorting technique risked affecting caspase-3 mRNA levels, RT-PCR was here performed on CD19+ MACS highly enriched splenic B cells. Figure 4C shows a significant increase in mRNA for caspase-3 (confirming the WB result presented in Fig. 4B) and down-regulation of mRNA levels for the anti-apoptotic Bcl-2 protein and BAFF-R 7 days post-infection. This pro-apoptotic gene regulation preceded the disappearance of the MZ B cells from the spleen.

### *T. brucei* infections abrogate *ex vivo* B cell proliferation

**Figure 5:**
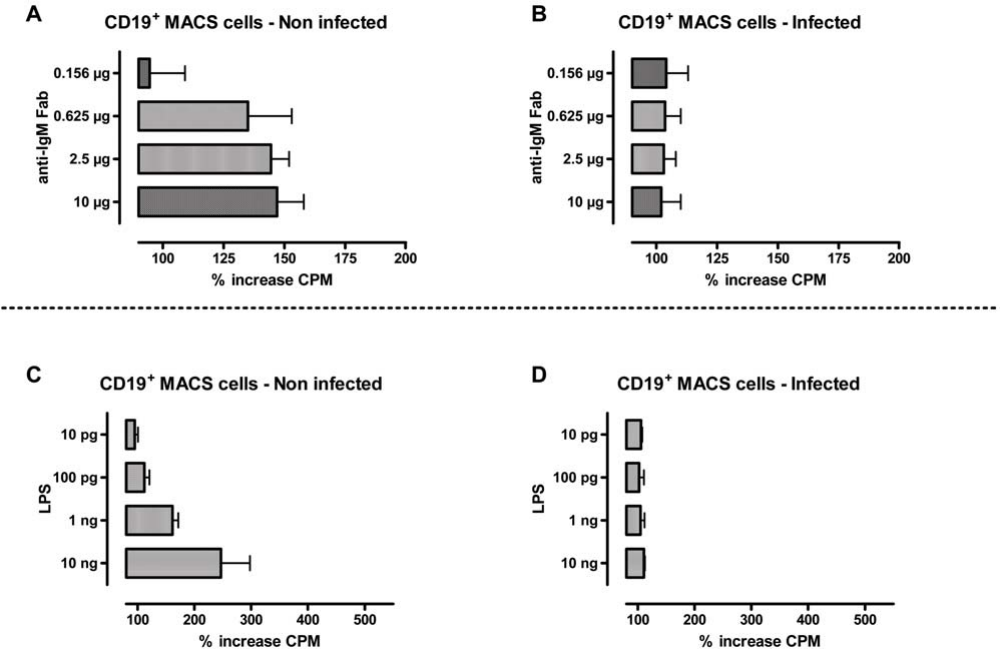
*T. brucei* induced abrogation of B cell proliferation. CD19^+^ MACS sorted cells derived form control mice or *T.brucei* AnTat 1.1E infected mice (day 10 post infection) were incubated for 24 h in the presence of different doses of anti-IgM Fab (A,B), or different doses of LPS (C,D). Proliferation was measure by thymidine incorporation. Results were obtained using spleen cell preparations of four individual mice, and represent the mean % of CPM increase ±SD, with the 100% showing the mean CPM level of non-stimulated cells.

The RT-PCR data presented above indicates that *T. brucei* infections induce apoptosis in populations of IgM^+^ spleen MZ B cells and MACS sorted CD19+ cells, hence, the later population was used to assess the effect of the infection on the IgM^+^B cell proliferative response. The sorted cells were incubated for 24 hours with different concentrations of anti-IgM Fab or LPS. Figure 5A indicates that naïve splenic B cells contain a cell fraction that is highly responsive to IgM Fab activation and proliferation. This fraction is absent in spleens of *T. brucei* infected C57Bl/6 mice 10 days after infection (Fig. 5B). Also when cells were stimulated in a non-specific manner with LPS, naïve CD19+ B cells showed a dose dependent proliferative response (Fig. 5C), while infection derived affected B cells showed a complete inhibition of LPS-mediated proliferation (Fig. 5D).

### The loss of IgM^+^ B cells and protective IgM responses renders mice susceptible to re-challenge with *T. brucei*

**Figure 6:**
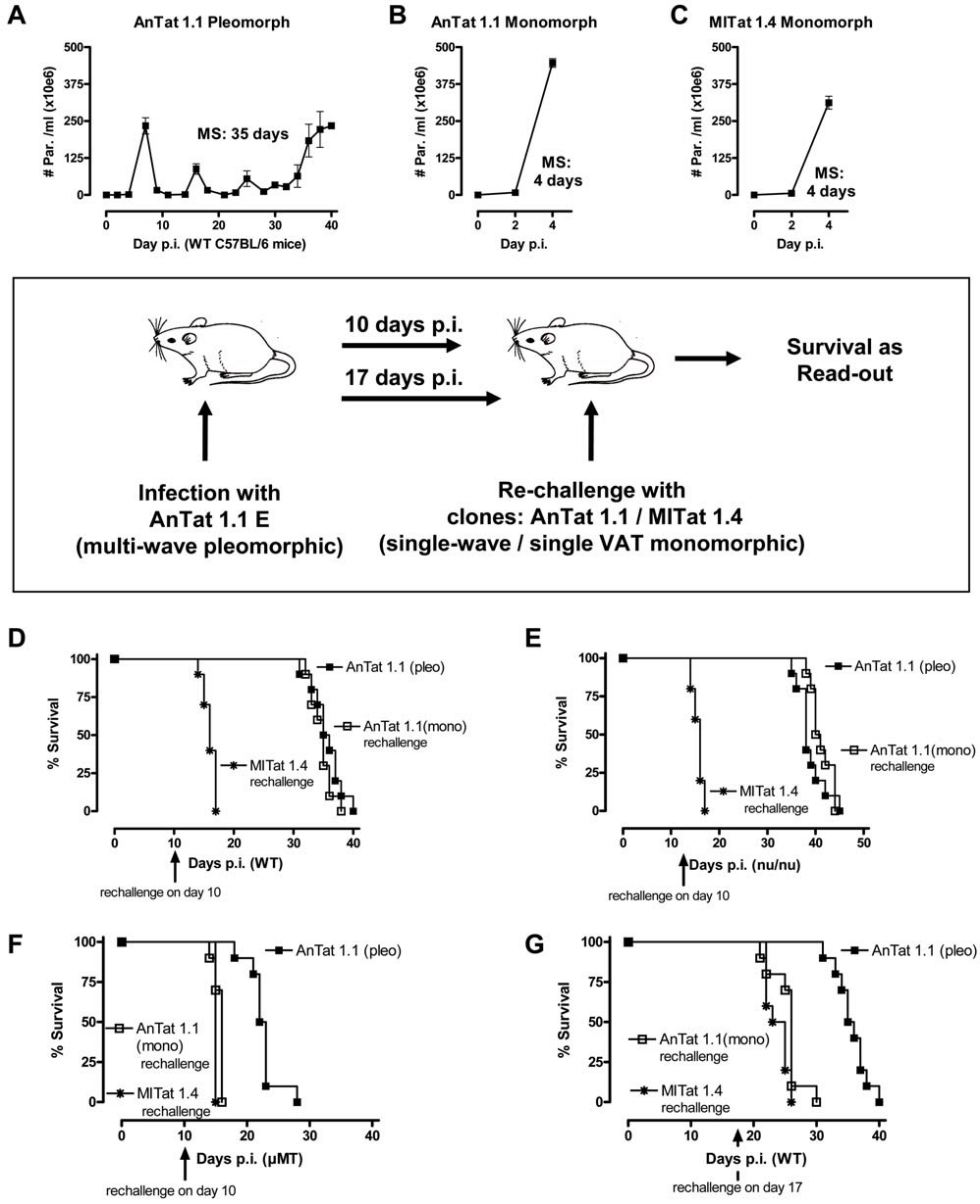
Trypanosomiasis associated elimination of parasite specific host antibody responses. Survival of mice was recorded after intra-peritoneal infection with of 5000 living parasites, using pleomorphic AnTat 1.1 (A), cloned monomorphic AnTat 1.1 (B), and unrelated cloned monomorphic MITat 1.4 (C) parasites (MS: Median survival). Re-challenge experiments were performed as presented in the insert box. Survival was recorded for the primary pleomorphic AnTat 1.1 infection (▪), and mice re-challenged with the cloned monomrophic AnTat 1.1 (□) or MiTat 1.4 (*) parasites on day 10, in WT mice (D), T-cell deficient nu/nu mice (E), and B-cell deficient µMT mice (F). A re-challenge was subsequently also performed on day 17 in WT mice (G). In each experiment 10 mice per experimental condition were used. One out of three representative experiments is shown for every experimental condition.

In order to analyze the consequence of the loss of B cell populations and in particular IgM^+^ B cells on the anti-trypanosome immune response, a series of re-challenge experiments were performed. Here, mice were infected with the pleomorphic AnTat 1.1 *T. brucei* parasite (that was used in all previous experiments presented in this paper) (Fig. 6A). The first VAT (VSG antigenic type) that emerges during this infection corresponds to the AnTat 1.1 VSG, while later parasitemia peaks correspond to subsequent VATs. In a separate experiment, mice were infected with the monomorphic AnTat 1.1 and MITat 1.4 parasites, representing clones that do not switch their VAT during their short and highly virulent infection (Fig. 6B/C). Next, infections were combined in order to analyze how mice react to a re-challenge with parasites expressing a previously encountered VAT.

The first re-challenge experiment was performed at day 10. This time point corresponds to the time point of infection in which infection-induced IgM^+^ plasma cells reach peak numbers (see Fig. 3), and is accompanied by peak levels of anti-trypanosome serum IgM titers that were previously shown to occur at day 10 [16]. Figure 6D shows that here a complete VAT/VSG-specific protection is obtained against the monomorphic AnTat 1.1 clone but not against the non-related MITat 1.4 parasite clone. This protection was retained in T-cell deficient nude mice (nu/nu) (Fig. 6E) but was absent in B-cell deficient µMT mice (Fig. 6F), suggesting that the protective immune response relies entirely on T-independent B-cell responses.

Next, a re-challenge experiment was done at day 17, corresponding to the time point where both IgM^+^MZ and IgM+ plasma B-cells were dramatically reduced. Here, both the AnTat 1.1 and MiTat 1.4 clones killed the mice rapidly (Fig. 6G). These results show that the AnTat 1.1 specific antibody mediated protection acquired by day 10 post-primary infection was already lost by day 17. A re-challenge experiment at day 24 confirmed the permanent loss of VSG-specific immunity (data not shown).

### *T. brucei* infection abrogates vaccine induced protection against a non-related pathogen such as *Bordetella pertussis*

**Figure 7:**
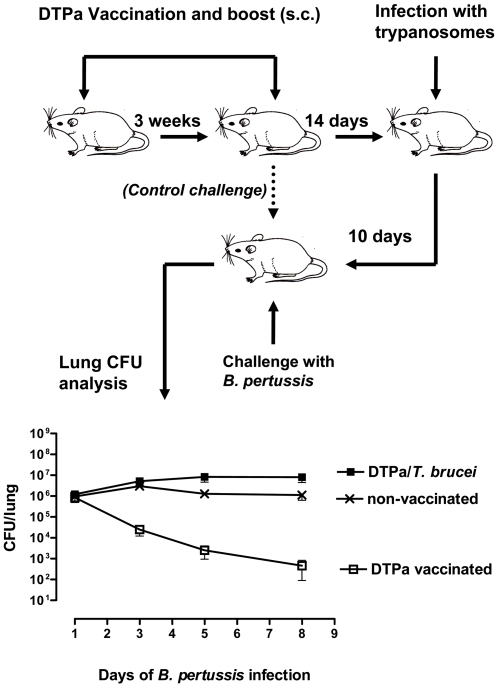
Trypanosomiasis associated elimination of non-related host antibody responses. Mice were vaccinated with the commercial DPTa vaccine and boosted after three weeks. 14 days after the vaccine boost, mice were infected with 5000 *T. brucei* AnTat 1.1E parasite by intra-peritoneal injection, followed 10 days later by an intranasal challenged with 5×10^6^ CFU of *B. pertussis/*mouse (▪). Control groups consisted of non-vaccinated *B. pertussis* challenged mice (×), and DPTa vaccinated mice that were challenged with *B. pertussis* 24 days after the second DTPa boost (□). Mice were sacrificed 3h and 3, 5 and 8, days after challenge and lung homogenates were prepared and plated on the Bordet-Gongou agar plates. CFU's were measured after 72 h incubation. Values are represented as the mean±SD of 3 individual mice per time point.

Following the finding that VAT-specific immunity is rapidly lost during trypanosome infections, we investigated whether *T. brucei* infection may have a general detrimental effect on the immune response generated by vaccination. As there is no efficient vaccine available against trypanosomes we tested whether *T. brucei* infection may abrogate the vaccine-induced protective immune response against a non-related pathogen such as *B. pertussis.* In order to do so, the commercially available DTPa vaccine was used. This vaccine protects children against infections such as *B. pertussis*, diphtheria and tetanus. The DTPa vaccination scheme has been well established and standardized in mice [36],[37]. It includes one vaccination and one booster after three weeks with the DTPa that leads to the induction of a specific protective antibody response. As indicated in Fig. 7, in contrast to non-vaccinated mice, DTPa vaccinated mice that were challenged with *B. pertussis* clear the bacteria from the lungs. However, this DTPa vaccine-mediated protective effect was abrogated in vaccinated mice that suffered a *T. brucei* infection prior to *B. pertussis* challenge. These results indicated that a *T. brucei* infection is capable of abrogating the efficacy of the vaccine-induced protective responses against non-related pathogens such as *B. pertussis*.

## Discussion

To date a large body of evidence is available addressing the molecular mechanisms underlying antigenic variation in African Trypanosomes. Antigenic variation is considered to be advantageous for the parasite as it permits continuous change of the surface antigenic coat and allows the parasites to evade antibody-mediated elimination [3],[7]. Here we demonstrate that in addition, trypanosome infections also cause the significant loss of various B cell populations and extensively remodel splenic micro-architecture. These changes (i) disable the hosts' capacity to mount a protective anti-parasite antibody responses, (ii) prevent the development of effective B-cell memory against encountered variant antigenic parasite types (VATs), and (iii) abrogate vaccine-induced protective responses to non-related pathogen such as *B. pertussis* in a setting where a commercially available human vaccine for diphtheria, tetanus and *B. pertussis* (DTPa) was used in an experimental mouse model.

Trypanosome infections are known to induce B cell unresponsiveness to mitogens and to induce polyclonal B-cell activation. In addition, highly virulent infections in which mice fail to control the first peak of infection have been shown to result in the rapid elimination of splenic FoB cells [31]. However, as natural trypanosome infections develop into a chronic phase, we analyzed here the fate of various splenic B cell populations throughout the course of an experimental *T. brucei* infection, using a more chronic, multi-wave pleomorphic model. In the spleen, B-cell populations can be subdivided into marginal zone (MZ) B cells, follicular (Fo) B cells, plasma (Pl) and germinal center B cells, as well as transitional T1 and T2 B cells [23],[24],[25],[26]. Our results first of all confirm previous data and show that in the early stage pleomorphic *T. brucei* infection extensive spleen remodeling gives rise to a rapid induction of spenomegaly due to cell proliferation and the influx of cells into the spleen [19],[38]. The detailed analysis of the splenic cell populations showed that following the first parasitemia peak mature B220^+^CD138^+^ plasma B cell numbers significantly increased, and that both IgM^+^ and IgM^−^ plasma cells accumulated at this stage. Later on in infection, B220^+^GL7^+^/PNA+ ‘germinal center’ B cells accumulated in the spleen as well, despite the absence of actual germinal center formation during trypanosome infections [40]. Interestingly, while ongoing trypanosome infections continuously generate new parasite antigentic types, major p1asma B cell induction occurs only at the beginning of infection in a response to the first encountered parasite VAT. Coinciding to this early plasma B cell induction, we recorded the total destructions of the spleen marginal zone (MZ), accompanied by the disappearance of the MZ B cell population, characterized as B220^+^IgM^High^IgD^Int^CD21^High^CD23^Low^ CD1d^+^CD138^−^ cells [26]. As the spleen marginal zone (MZ) separates the T- and B-cell containing white pulp from the blood filled sinuses of the red pulp, the MZ is involved in the capture of blood-born pathogens, the regulation of lymphocyte trafficking, and the induction of antigen specific T-independent B cell responses, mostly resulting in IgM secretion [23],[24]. Important here is that previously published data indicated that T-independent VSG specific IgM responses are crucial in trypanosome clearance during ongoing infection [16],[17],[22]. Using a VSG-specific re-challenge model, we now show that the infection-associated destruction of the IgM^+^ MZ B cell compartment results in the rapid loss of IgM-mediated VSG specific protection against re-infection with a previously encountered parasite.

The infection-associated disappearance of the MZ B cells from the spleen could be explained by two independently occurring mechanisms, being cell differentiation and/or cell death. Supporting the first is the observation that the rapid disappearance of MZ B cells coincided with the temporary accumulation of IgM^+^ plasma cells. However, a direct link between these two events remains speculative, as no specific markers exist that allow cell fate analysis that would proof the MZ B cell origin of the occurring plasma cells. Hence, in order to address this possibility, we aimed at reconstituting JH^−/−^ B-cell deficient mice with naïve IgM^+^ B cells. During *T. brucei* infections however, this approach did not result in efficient B cell repopulation or the accumulation of IgM^+^ plasma cells (unpublished results S. Black). On the other hand, our results suggest that the rapid disappearance of MZ B cells does involve the induction of parasitemia-associated B-cell apoptosis. Indeed analysis of the CD21^High^CD23^Low^ MZ B cells that remain in the spleen in the days following the clearance of the first peak of parasitemia, revealed that these cells upregulated Annexin V expression and in large stained positive for 7AAD. In addition, the induction of caspase 3 gene expression as well as the conversion of pro-caspase 3 into the cleaved 12 kD and 17 kD caspase 3 activation products in this cell population, suggests the induction of trypanosomiasis-associated apoptosis in the splenic MZ B cell population. Moreover, the affected MZ B cells showed down-regulation of mRNA for the anti-apoptotic Bcl-2 protein, involved in the inhibition of caspase 3 activation through the cytochrome C pathway [28]. Finally, also the mRNA expression for the B cell specific BAFF-R was reduced in the vanishing MZ B cell population. This receptor normally governs B cell homeostasis and provides an NF-κB activation signal that regulates the mRNA levels for the anti-apoptotic Bcl2 protein [27],[28]. Interesting to note here is that parasite-induced B-cell apoptosis has previously also been reported to occur in experimental infections with the intracellular *T. cruzi* parasite. In this case however, the process was mediated by Fas/Fas Ligand interactions resulting in B cell-B cell fratricide, selectively affecting IgG^+^ B-cells [41].

In an independent approach, *T. brucei* induced spleen dysfunction was assessed by immunohistochemistry. Here we show the profound destruction of splenic MZ micro-architecture is marked by the loss of MZM and MMM (macrophage) populations. Detailed analysis of the spleen remodeling, revealed that beside the loss of MZ architecture, the entire spleen structure encompassing white and red pulp is lost, showing the absence of germinal center formation. This destruction of splenic microarchitecture occurred during the first peak of infection, and was permanent. Due to the destruction of the spleen microarchitecture in combination with (i) the alteration of splenic B cell surface markers, and (ii) the drastic decrease in particular B cell subsets, immunohistochemistry could unfortunately not be used for the co-localization of apoptotic markers and specific MZ B cell markers. Important to note here is that remodeling of spleen cell architecture has also been reported in other experimental parasite infection models. Indeed, experimental *Plasmodium chabaudi* infections in mice also cause major disruptions in the splenic cell distribution as well as the splenic micro-architecture. However, as this infection is self-curing, in contrast to experimental trypanosome infections, these changes were found to be temporary [42].

The last B cell aspect presented in this paper, deals with the question of whether *T. brucei* infection can actively abrogate a vaccine-induced protective memory immune response. This point is extremely relevant to address in the context of multiple ongoing vaccination programs in the human African population such as the recent WHO Meningitis Vaccine Project (MVP), and the Pediatric Dengue Vaccine Initiative (PDVI), and in the context of future anti- HIV/AIDS and anti-malaria vaccine programs. To address this question, we applied the commercially available human DTPa vaccine against diphtheria, tetanus and *B. pertussis* in a *T. brucei* mouse model [36],[37]. While our findings showed the expected protective vaccine response in control vaccinated and *B. pertussis* challenged mice, the DTPa efficacy was abolished after the vaccinated host was infected with *T. brucei* parasites. These results indicate that the ongoing trypanosome infection either depleted the memory B cell compartment that was first generated during the DTPa vaccination, or prevented the reactivation of the memory response. Taking into account that the commercially available DTPa vaccine [37] is routinely used in Africa and other countries to protect children against diphtheria, tetanus, and *B. pertussis*, our results raise the worrying possibility that the protective potential of this vaccine can be abolished by a later encounter with *T. brucei* parasites, exposing the individual to secondary infection hazards and abolishing previous health efforts. In this context it is remarkable to note that studies performed 3 decades ago already indicated that *T. brucei* infections abrogate B cell memory responses to thymus-dependent and thymus-independent antigens using DNP-KLH and DNP-Ficoll as antigens for vaccination [29]. Additionally, earlier investigations in domestic animals showed that infections with African trypanosomes abrogate the efficacy of several commercial vaccines against the foot and mouth disease, swine fever, anthrax spores, *Brucella abortis* and louping-ill vaccine [43],[44],[45],[46],[47]. However, till now no attention has been given to the consequences of *T. brucei* infections on the immune response induced by commercially available human vaccines. In addition, these findings have not been seriously considered in recent years when (failed) anti-trypanosome vaccination efforts were reported. Indeed several attempts have now been made to develop anti-trypanosome vaccines using relatively non-variant trypanosome antigens, but none of these attempts have been successful so far, despite the initial induction of antigen-specific antibody responses [8],[48],[49],[50]. In view of the results presented here, it is possible that the rapid trypanosome driven B-cell dysfunction upon host contact with living parasites contributed to these vaccine failures. Our studies suggest that only vaccines that strongly suppress the level of initial parasitemia peak or induce immediate sterile immunity would be likely to have sufficient efficacy in trypanosomiasis susceptible hosts. In addition, it is crucial to realize that due to the constraints of conventional strategies, most vaccination protocols lead to the induction of T-dependent high affinity IgG responses. Taking into account that the protective response against previously encountered VAT present in ‘day 10’ re-challenged mice was T-independent and IgM-mediated, this poses an additional obstacle for vaccine development, as till now no strategies exist to generate long lasting high affinity IgM responses.

## References

[1] Vickerman K (1989). Trypanosome sociology and antigenic variation.. Parasitology.

[2] Vickerman K (1978). Antigenic variation in trypanosomes.. Nature.

[3] Pays E (2006). The variant surface glycoprotein as a tool for adaptation in African trypanosomes.. Microbes Infect.

[4] Pays E (2005). Regulation of antigen gene expression in Trypanosoma brucei.. Trends Parasitol.

[5] Borst P (2002). Antigenic variation and allelic exclusion.. Cell.

[6] Taylor JE, Rudenko G (2006). Switching trypanosome coats: what's in the wardrobe?. Trends Genet.

[7] Pays E (1995). [Antigenic variation and the problem of vaccines against African trypanosomes].. Bull Mem Acad R Med Belg.

[8] Hertz CJ, Mansfield JM (1999). IFN-gamma-dependent nitric oxide production is not linked to resistance in experimental African trypanosomiasis.. Cell Immunol.

[9] Magez S, Radwanska M, Beschin A, Sekikawa K, De Baetselier P (1999). Tumor necrosis factor alpha is a key mediator in the regulation of experimental Trypanosoma brucei infections.. Infect Immun.

[10] Radwanska M, Magez S, Dumont N, Pays A, Nolan D (2000). Antibodies raised against the flagellar pocket fraction of Trypanosoma brucei preferentially recognize HSP60 in cDNA expression library.. Parasite Immunol.

[11] Levine RF, Mansfield JM (1984). Genetics of resistance to the African trypanosomes. III. Variant-specific antibody responses of H-2-compatible resistant and susceptible mice.. J Immunol.

[12] Van Meirvenne N, Magnus E, Buscher P (1995). Evaluation of variant specific trypanolysis tests for serodiagnosis of human infections with Trypanosoma brucei gambiense.. Acta Trop.

[13] Vincendeau P, Daulouede S, Veyret B, Darde ML, Bouteille B (1992). Nitric oxide-mediated cytostatic activity on Trypanosoma brucei gambiense and Trypanosoma brucei brucei.. Exp Parasitol.

[14] Black SJ, Hewett RS, Sendashonga CN (1982). Trypanosoma brucei variable surface antigen is released by degenerating parasites but not by actively dividing parasites.. Parasite Immunol.

[15] Reuner B, Vassella E, Yutzy B, Boshart M (1997). Cell density triggers slender to stumpy differentiation of Trypanosoma brucei bloodstream forms in culture.. Mol Biochem Parasitol.

[16] Radwanska M, Magez S, Michel A, Stijlemans B, Geuskens M (2000). Comparative analysis of antibody responses against HSP60, invariant surface glycoprotein 70, and variant surface glycoprotein reveals a complex antigen-specific pattern of immunoglobulin isotype switching during infection by Trypanosoma brucei.. Infect Immun.

[17] Reinitz DM, Mansfield JM (1990). T-cell-independent and T-cell-dependent B-cell responses to exposed variant surface glycoprotein epitopes in trypanosome-infected mice.. Infect Immun.

[18] Baral TN, De Baetselier P, Brombacher F, Magez S (2007). Control of Trypanosoma evansi infection is IgM mediated and does not require a type I inflammatory response.. J Infect Dis.

[19] Diffley P (1983). Trypanosomal surface coat variant antigen causes polyclonal lymphocyte activation.. J Immunol.

[20] Oka M, Ito Y, Furuya M, Osaki H (1984). Trypanosoma gambiense: immunosuppression and polyclonal B-cell activation in mice.. Exp Parasitol.

[21] Buza J, Naessens J (1999). Trypanosome non-specific IgM antibodies detected in serum of Trypanosoma congolense-infected cattle are polyreactive.. Vet Immunol Immunopathol.

[22] Dubois ME, Demick KP, Mansfield JM (2005). Trypanosomes expressing a mosaic variant surface glycoprotein coat escape early detection by the immune system.. Infect Immun.

[23] Lopes-Carvalho T, Foote J, Kearney JF (2005). Marginal zone B cells in lymphocyte activation and regulation.. Curr Opin Immunol.

[24] Song H, Cerny J (2003). Functional heterogeneity of marginal zone B cells revealed by their ability to generate both early antibody-forming cells and germinal centers with hypermutation and memory in response to a T-dependent antigen.. J Exp Med.

[25] Sagaert X, Sprangers B, De Wolf-Peeters C (2007). The dynamics of the B follicle: understanding the normal counterpart of B-cell-derived malignancies.. Leukemia.

[26] Gorelik L, Cutler AH, Thill G, Miklasz SD, Shea DE (2004). Cutting edge: BAFF regulates CD21/35 and CD23 expression independent of its B cell survival function.. J Immunol.

[27] Schneider P (2005). The role of APRIL and BAFF in lymphocyte activation.. Curr Opin Immunol.

[28] Rahman ZS, Manser T (2004). B cells expressing Bcl-2 and a signaling-impaired BAFF-specific receptor fail to mature and are deficient in the formation of lymphoid follicles and germinal centers.. J Immunol.

[29] Askonas BA, Corsini AC, Clayton CE, Ogilvie BM (1979). Functional depletion of T- and B-memory cells and other lymphoid cell subpopulations-during trypanosomiasis.. Immunology.

[30] Clayton CE, Selkirk ME, Corsini CA, Ogilvie BM, Askonas BA (1980). Murine trypanosomiasis: cellular proliferation and functional depletion in the blood, peritoneum, and spleen related to changes in bone marrow stem cells.. Infect Immun.

[31] Sacco RE, Hagen M, Donelson JE, Lynch RG (1994). B lymphocytes of mice display an aberrant activation phenotype and are cell cycle arrested in G0/G1A during acute infection with Trypanosoma brucei.. J Immunol.

[32] Thompson CB (1995). Apoptosis in the pathogenesis and treatment of disease.. Science.

[33] Kerr JF, Wyllie AH, Currie AR (1972). Apoptosis: a basic biological phenomenon with wide-ranging implications in tissue kinetics.. Br J Cancer.

[34] Boatright KM, Salvesen GS (2003). Mechanisms of caspase activation.. Curr Opin Cell Biol.

[35] Li Y, Wang B, Zhou C, Bi Y (2007). Matrine induces apoptosis in angiotensin II-stimulated hyperplasia of cardiac fibroblasts: effects on Bcl-2/Bax expression and caspase-3 activation.. Basic Clin Pharmacol Toxicol.

[36] Denoel P, Godfroid F, Guiso N, Hallander H, Poolman J (2005). Comparison of acellular pertussis vaccines-induced immunity against infection due to Bordetella pertussis variant isolates in a mouse model.. Vaccine.

[37] Roduit C, Bozzotti P, Mielcarek N, Lambert PH, del Giudice G (2002). Immunogenicity and protective efficacy of neonatal vaccination against Bordetella pertussis in a murine model: evidence for early control of pertussis.. Infect Immun.

[38] Igbokwe IO, Nwosu CO (1997). Lack of correlation of anaemia with splenomegaly and hepatomegaly in Trypanosoma brucei and Trypanosoma congolense infections of rats.. J Comp Pathol.

[39] Benedict CA, De Trez C, Schneider K, Ha S, Patterson G (2006). Specific remodeling of splenic architecture by cytomegalovirus.. PLoS Pathog.

[40] Morrison WI, Murray M, Bovell DL (1981). Response of the murine lymphoid system to a chronic infection with Trypanosoma congolense. I. The spleen.. Lab Invest.

[41] Zuniga E, Motran CC, Montes CL, Yagita H, Gruppi A (2002). Trypanosoma cruzi infection selectively renders parasite-specific IgG+ B lymphocytes susceptible to Fas/Fas ligand-mediated fratricide.. J Immunol.

[42] Achtman AH, Khan M, MacLennan IC, Langhorne J (2003). Plasmodium chabaudi chabaudi infection in mice induces strong B cell responses and striking but temporary changes in splenic cell distribution.. J Immunol.

[43] Whitelaw DD, Scott JM, Reid HW, Holmes PH, Jennings FW (1979). Immunosuppression in bovine trypanosomiasis: studies with louping-ill vaccine.. Res Vet Sci.

[44] Sharpe RT, Langley AM, Mowat GN, Macaskill JA, Holmes PH (1982). Immunosuppression in bovine trypanosomiasis: response of cattle infected with Trypanosoma congolense to foot-and-mouth disease vaccination and subsequent live virus challenge.. Res Vet Sci.

[45] Rurangirwa FR, Musoke AJ, Nantulya VM, Tabel H (1983). Immune depression in bovine trypanosomiasis: effects of acute and chronic Trypanosoma congolense and chronic Trypanosoma vivax infections on antibody response to Brucella abortus vaccine.. Parasite Immunol.

[46] Mwangi DM, Munyua WK, Nyaga PN (1990). Immunosuppression in caprine trypanosomiasis: effects of acute Trypanosoma congolense infection on antibody response to anthrax spore vaccine.. Trop Anim Health Prod.

[47] Holland WG, Do TT, Huong NT, Dung NT, Thanh NG (2003). The effect of Trypanosoma evansi infection on pig performance and vaccination against classical swine fever.. Vet Parasitol.

[48] Paling RW, Moloo SK, Scott JR, Gettinby G, McOdimba FA (1991). Susceptibility of N'Dama and Boran cattle to sequential challenges with tsetse-transmitted clones of Trypanosoma congolense.. Parasite Immunol.

[49] Mkunza F, Olaho WM, Powell CN (1995). Partial protection against natural trypanosomiasis after vaccination with a flagellar pocket antigen from Trypanosoma brucei rhodesiense.. Vaccine.

[50] Authie E, Boulange A, Muteti D, Lalmanach G, Gauthier F (2001). Immunisation of cattle with cysteine proteinases of Trypanosoma congolense: targetting the disease rather than the parasite.. Int J Parasitol.

